# Optical Coherence Tomography Velocimetry for In-Line Processing of Biologics: Concentrated and Gelling Monoclonal Antibody Solutions

**DOI:** 10.1021/acsengineeringau.5c00083

**Published:** 2026-02-24

**Authors:** Conor M. Lewis, Owen Watts Moore, Charles T. Heise, Jennifer Tovey, Thomas A. Waigh

**Affiliations:** † Biological Physics Group, Department of Physics and Astronomy, 5292 University of Manchester, Oxford Road, Manchester M13 1LP, U.K.; ‡ FUJIFILM Biotechnologies UK Ltd, Billingham, England TS23 1LH, U.K.

**Keywords:** monoclonal antibody, downstream purification, optical coherence tomography, velocimeter, bovine serum albumin, thixotropic

## Abstract

Optical coherence tomography velocimetry (OCTV) was demonstrated with in-line processing of biologics for the first time. OCTV allowed the velocity of concentrated monoclonal antibodies (mAbs at 39.5–84.7 mg mL^–1^) to be probed in 3.4 pL volumes over distances 0–5 mm from the pipe walls. The large penetration depth is facilitated by the relatively low turbidity of mAbs at near-infrared wavelengths (1300 nm). The mAb solutions could be concentrated in situ and the changes to the viscoelasticity measured. Higher concentration mAb solutions became shear thinning (following the power law fluid model) and the amplitude of their velocity fluctuations decreased. Furthermore, dropping the pH of the mAb solutions induced a gelation phase transition and complex changes to the mAb rheology could be observed with OCTV e.g. thixotropy and the formation of a stationary boundary layer. Thus, in situ formulation of mAbs could be explored with OCTV under industrially relevant conditions.

## Graphical abstract

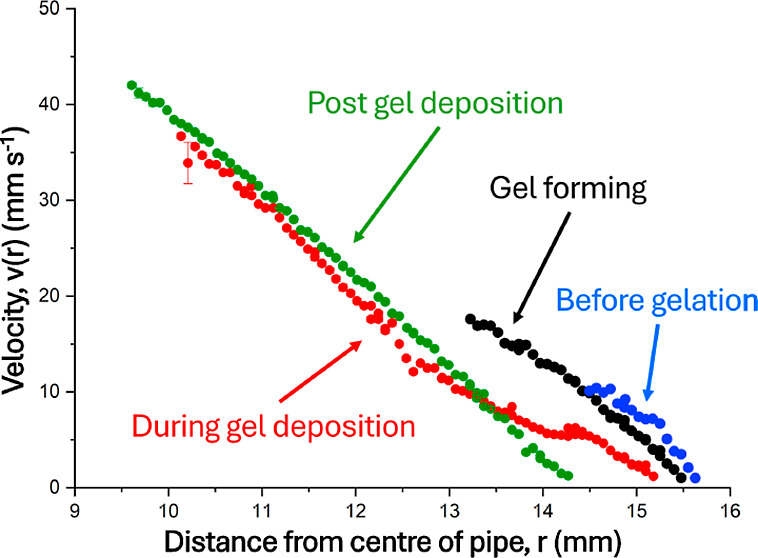

## Introduction

Monoclonal antibodies (mAbs) are recombinant therapeutic proteins with a wide range of uses in modern medicine, such as for the treatment of autoimmune diseases (e.g., rheumatoid arthritis),^1^ cancer immunotherapy (e.g., checkpoint inhibitors like nivolumab and pembrolizumab),^2^ and antigen tests for infectious diseases like COVID-19.^3^ During large-scale purification in downstream processing, mAb solutions typically circulate through a semicontinuous flow path.^4^ For instance, in protein A chromatography, mAbs are pumped through a column until fully adsorbed to the resin, after which flow is paused, an elution buffer is introduced, and the mAbs are flushed from the column.^5^ The SymphonX system is designed to integrate downstream purification operations within a single unit.^6^ A key objective of this platform is to enable real-time, in-line monitoring of critical process parameters to optimize yield and reduce operational costs.^7,8^ The integrated sample analysis chamber includes pH, conductivity, and UV–vis probes calibrated for dynamic flows, offering immediate feedback during processing.

However, several critical mAb characteristics, such as precise concentration and aggregation analysis, are still assessed offline. In particular, accurate flow profiling and pressure monitoring are essential for processes like tangential flow filtration and chromatographic optimization. This manuscript explores the use of optical coherence tomography velocimetry (OCTV) as a noninvasive, real-time analytical technique suited for in-line characterization of mAb flows. The technique offers robust performance in optically dense (e.g., turbid), scattering environments. Novel data is presented, capturing concentration and shear-dependent power-law behavior, and deposition dynamics and shear dependent viscosity during gelation of a mAb. The applicability of OCTV in flow regimes relevant to downstream processing is evaluated and discussed in detail.

While this OCTV apparatus has been previously demonstrated in opaque lamellar surfactant gel networks and cloudy milk solutions,^9^ and other devices using dense polymer melts,^10^ its function in dilute and relatively transparent biologic suspensions has not. Here, we apply OCTV to biologic processing for the first time and demonstrate its utility with both mAbs and bovine serum albumin (BSA).

The bulk viscoelasticity of concentrated mAb solutions was measured previously by our group using particle tracking microrheology (PTM).^11,12^ The mAb rheology follows that expected for electrostatically stabilized Brownian colloids e.g. a modified Mooney relationship could describe the viscosity as a function of mAb concentration.^11^ Addition of acid to the mAb solutions could induce a continuous gelation phase transition, but it was sensitive to the variety of mAb chosen (mAb-2 was sensitive whereas mAb-1 was not). Furthermore, PTM and dynamic light scattering experiments showed the data was in agreement with dynamic scaling models for the fractal aggregates of mAbs in the gels. The process of gelation appeared to occur via amyloid formation with evidence from Thioflavin T binding and circular dichroism. The reduction of pH is directly relevant to industrial mAb formulation because it is used to inactivate viruses and remove the mAbs from chromatography columns.

## Methods

Two mAbs, both supplied by FUJIFILM Diosynth Biotechnologies, were investigated in this study. The first, designated mAb-1, consists of an IgG1 heavy chain paired with a lambda-III light chain and has a storage buffer of 20 mM sodium phosphate pH 6.0, 0.01% (w/v) polysorbate 20 and 7.5% sucrose. The second, mAb-2, also features an IgG1 heavy chain but is paired with a kappa-I light chain, in a buffer of 50 mM sodium phosphate pH 7.5, 0.01% (w/v) polysorbate 20. Both mAbs had similar molecular weights of 144 kDa. A bovine serum albumin solution was also tested (see Supporting Information) that was diluted in identical buffer to mAb-1 to a concentration of 40 mg mL^–1^.

The apparatus, detailed in Figure 1, is based on a Mach–Zehnder interferometer^13^ in which light from a broadband superluminescent diode (SLD) is split between a reference and sample arm through optic fiber couplers.^14,15^ The reference arm contains an optical delay line holding a mirror on a controllable translation stage which reflects back through the fiber optic cables. The sample arm light is collimated and focused through two lenses and reflected off the sample which contains flowing antibodies in a pipe section. The light is recombined at the detector where the coherence gate allows small volume sample scanning (sensitive to 3.4 pL volumes). The super luminescent diode light source has a wavelength of λ = 1300 nm and a bandwidth of Δλ = 65 nm, giving a coherence length of *l*_c_ = 9 μm. Coherence gating^14^ allows for *l*_c_ resolution of scanning, with depth in the pipe i.e. the OCTV technique can resolve slices within the sample of thickness *l*_c_. The position in the pipe is adjusted via the movement of the mirror in the translation stage, accounting for the refractive index of the fluid and angle of the lens.

**Figure 1:**
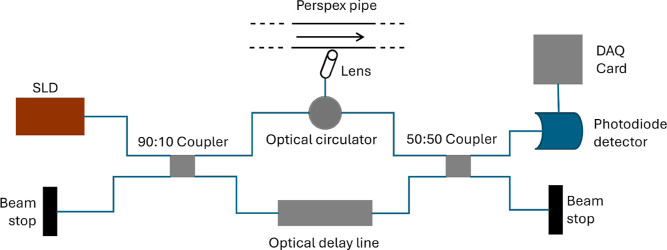
Schematic diagram of the OCT device based on a Mach–Zehnder interferometer.^9^ Light is split from a broadband super luminescent diode between a reference and sample arm and interferes at the detector without traveling the same path. Single mode angled optical fibers carry the light into a lens incident on a perspex pipe section containing a flowing sample, where the Doppler frequency and velocity can be deduced at depths into the pipe.

A photodetector and high sample rate data acquisition card digitizes the signal and a LabVIEW program is used for control and data analysis. The interference pattern caused by the Doppler shift of moving samples is converted into the frequency domain via a power spectral density transformation. A Gaussian distribution can be fit to the Doppler frequencies and the velocity, *v*, of the sample can be calculated from the mean frequency

1 $$v=\frac{\lambda f_{D}}{2\;\sin\theta }$$

where λ is the wavelength of the SLD, *f*_D_ is the Doppler frequency at the Gaussian center and θ is the angle of the lens to the sample. A small angle of θ = 9.66° is employed to avoid surface backscattering which would otherwise dominate the Doppler shifted signal.

The flow path used in the SymphonX rig from FUJIFILM Diosynth Biotechnologies was specifically configured to support selected downstream purification processes relevant to this study. Components not directly involved in the experiments are omitted for clarity. A simplified schematic is shown in Figure 2. Fluid was circulated using a Quattroflow 1200-SU pump, which operates via a four-piston diaphragm mechanism and supports flow rates up to 1200 L h^–1^. The pump speed is controlled through an integrated panel and set as a percentage of the total pump capacity, adjustable from 0.1 to 100%. The majority of the system uses flexible silicone tubing with an internal radius of *r* = 6.35 mm. An in-line sample analysis chamber, fitted with both a pH and UV–vis probe, is integrated into the flow path. Due to the physical dimensions of these probes, the internal radius of the tubing within this chamber is expanded to *r* = 16.0 mm, with conical diffusers on either end to transition between the differing pipe diameters. Initially the OCTV probe was designed with the larger pipe specifications and thus also has an internal radius of *r* = 16.0 mm. To mitigate flow disturbances caused by this expansion, an additional length of large-radius piping was fitted on either side of the lens. The length from the end of the first diffuser to the lens totalled 220 mm.

**Figure 2:**
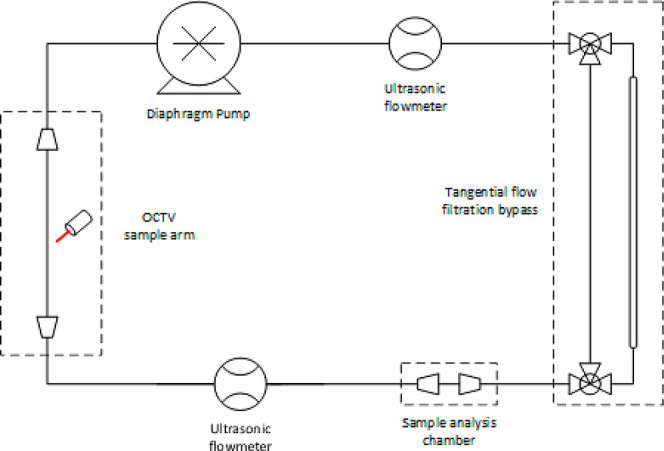
Simplified pipe and instrumentation diagram of the flow-path employed within the SymphonX rig. The difference in pipe radius from *r* = 6.35 mm to *r* = 16.0 mm is denoted by conical diffusers. Pipe length not to scale.

Two SONOFLOW CO.55 V2.0 ultrasonic flow meters were clamped to the outside of the silicone piping before and after the sample analysis chamber, which were used as a comparison for flow rate calculations. A bypass-valve was attached to a tangential flow filtration column to concentrate solutions using the differential pressure. Here the membrane was of sufficient size to only allow particles smaller than the mAb through. The total hold-up volume of the pipeline was 1.8 L when including the tangential flow filtration column, and 1.5 L when bypassed.

## Results and Discussion

### Flow of Low Concentration mAb-1 Solutions

The flow path was initialized with a flush of deionized water to the total hold-up volume, followed by pumping 3.8 L of a mAb-1 solution in its storage buffer. The final concentration of the mAb-1 solution was measured to be 39.04 ± 0.03 mg mL^–1^ using an off-line SoloVPE, where a variable path length was compared to multiple absorbance data points. The pump was then set to several fixed speeds as a function of power percentage, from 5 to 20% or 60–240 L h^–1^. The OCTV apparatus was used to take velocity measurements via identification of Doppler shifted peaks in the power spectral density of back scattered light. A cutoff frequency was required to avoid fitting the flicker noise (1/*f*) associated with the detector,^16^ effectively limiting the low velocity measurements of the device. The device was set to take ∼100 measurements at each point over 10 s, moving 100 μm in the delay line for each measurement. Accounting for the refractive index of *n* = 1.32 and angle of refraction as θ_r_ = 7.19°, this equaled sizes of 77 μm within the sample. The refractive index at 1300 nm was calculated from the following relationship^17,18^

2 $$n=n_{0}+c\left(\frac{dn}{dc}\right)$$

where *n*_0_ is the refractive index of the buffer solution, *c* is the mAb concentration and $\frac{dn}{dc}$ is the refractive index increment, calculated from the weights of amino acid composition in the mAb. The difference from the refractive index of water for wavelengths of 1300 nm, *n* = 1.31, is small in this case.^18^

The velocity measurements as a function of *r* are plotted in Figure 3. In a pipe flow where the fluid is incompressible, Newtonian and laminar across a pipe diameter smaller than the total length of the pipe, the velocity profile *v*(*r*) of radius *r* from the center of the pipe is well described by the Hagen–Poiseuille equation^19^ as derived from the Navier–Stokes equation

3 $$v\left(r\right)=\frac{\partial P}{\partial z}\frac{1}{4\mu }\left(R^{2}-r^{2}\right)$$

where $\frac{\partial P}{\partial z}$ is the change in pressure over a length *z*, μ is the dynamic viscosity and *R* is the radius of the pipe. This equation is fitted to all pump speeds in Figure 3 where $\frac{\partial P}{\partial z}\frac{1}{4\mu }$ is treated as a single constant, without a method to experimentally measure the pressure change, ∂P. The results of the mAb-1 solution are similar to that of an equally concentrated bovine serum albumin, shown in the Supporting Information.

**Figure 3:**
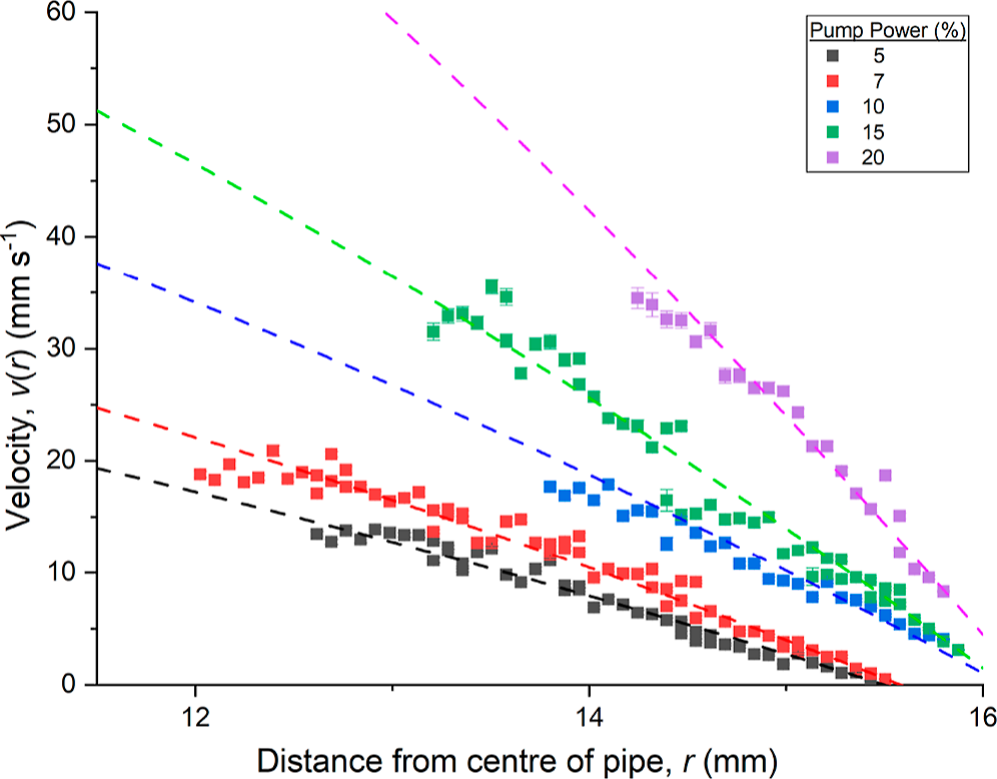
Velocity profile *v*(*r*) as a function of radial depth into the pipe section, *r* of a 39 mg mL^–1^ mAb-1 solution at different pump speeds as a percentage of total pump power. Dashed lines correspond to a fit of eq 3.

The dilute mAb-1 solution exhibits weak scattering intensity compared to turbid lamellar surfactant solutions^9^ due to the presence of smaller and less dense scatterers, resulting in reduced spatial resolution and broader Gaussian distributions in the power spectral density. This, combined with lower signal-to-noise ratios, contributes to increased stochastic fluctuations in the extracted velocity profiles. The residuals from the Hagen–Poiseuille fits are notably larger compared to those obtained for higher viscosity, optically dense fluids previously characterized using OCTV.^9^

For the 5 and 7% pump powers, quiescent layers were observed at 600 and 700 μm, respectively. These layers are likely a result of the redeveloping, low Reynolds number flow after the conical diffuser, where recirculation zones or near-wall regions may form. Similarly, Hagen–Poiseuille fits of the faster pump speeds (15 and 20% pump power) yield a pipe radius greater than the actual size of the pipe. When forced to obey this limit, the plot fails to accurately account for the remaining data points. Instead flow instability likely leads to turbulence and a viscous sublayer forms close to the pipe wall. Without low velocity measurements, these two regions are difficult to quantify exactly.

The penetration depth into the mAb sample is considerably higher than opaque complex fluids studied previously (e.g., conditioner and shampoo),^10,20^ extending 4 mm into the sample at 7% pump speed. The penetration depth also depends on the pump speed, with decreases seen in maximum penetration depth about the 7% measurement. The measurement depth limit is determined by the point where the Doppler frequency amplitude becomes indistinguishable from the background noise. As a result, the maximum depth is constrained by both the background noise and factors that reduce the amplitude of the detector signal. In opaque fluids, the contributions from multiple scattering and optical attenuation increase at a much higher rate with depth compared to those in semidilute mAb solutions.

These findings are verified with the statistical analysis shown in Figure 4. Here probability density functions (pdf) are formed through calculating $\frac{v-\mathrm{v̅}}{\mathrm{v̅}}$ at different lengths into the pipe and at different percentages of pump power. Each pdf had *N* ≈ 100 individual velocity measurements and the standard deviation, skew and kurtosis are calculated from the distribution. The standard deviation results show limited uniformity across pump power levels, with the lowest values observed at 10% pump power, followed by an increasing deviation at both higher and lower pump speeds, forming a *U*-shaped pattern. Variation is seen in the skewness and kurtosis, with the most precise values for all three statistical properties occurring at 20% pump speed. The standard deviations are relatively large across all pump speeds, particularly when compared to denser lamellar gel networks of surfactants,^9^ indicating greater stochastic fluctuations in velocity. This statistical variance may suggest the presence of intermittent turbulent flow leading to a deviation from Gaussian statistics.^21,22^

**Figure 4:**
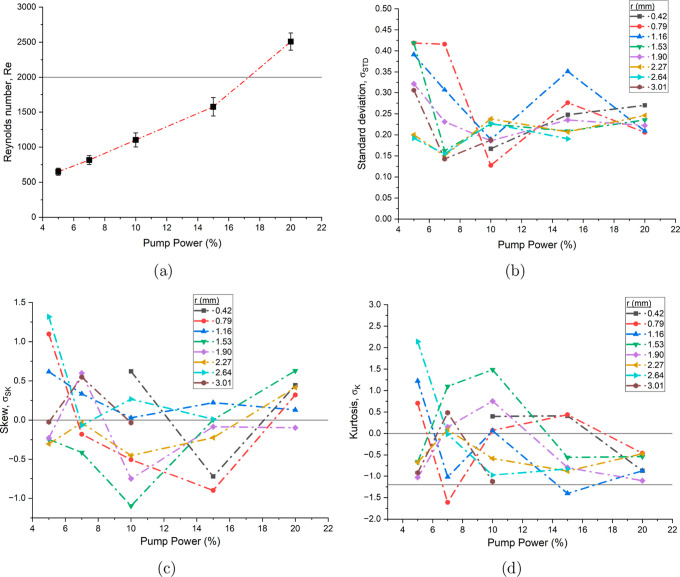
(a) Reynolds number, *Re*, as a function of pump power for a 39 mg mL^–1^ mAb-1 solution. Standard deviation (b), skew (c) and kurtosis (d) of probability density functions of $\frac{v-\mathrm{v̅}}{\mathrm{v̅}}$ as a function of pump power at different positions in the pipe for the same solution. Lines at *Re* = 2000 indicate the start of the transitional regime, σ_SK_ = 0 for zero skew, σ_K_ = 0 for a normal distribution and σ_K_ = −1.2 for a uniform distribution have been added to aid the viewer.

The Reynolds number is a dimensionless quantity and represents the ratio of inertia to viscosity, or the fluid’s turbulence.^23^ For a pipe flow, the Reynolds number (*Re*) is

4 $$Re=\frac{\rho Dv}{\eta }$$

where $v=\frac{1}{2}v_{\max}$ is the characteristic velocity, ρ is the density of the fluid, *D* = 2*R* is the characteristic length scale and η is the viscosity of the solution. Off-line microrheology^11^ experiments reveal a viscosity of η = 1.10 ± 0.04 mPa s for a 39 mg mL^–1^ mAb-1 solution, which will be used for calculations. The Reynolds number for different pump powers are shown in Figure 4a. Reynolds numbers below 2000 indicate steady laminar flow, while values above 2000 signal a transitional regime toward turbulence, where intermittent and localized turbulent regions exist within an otherwise laminar flow.^24^

The variance of the fluid velocities should be small in the dilute mAb-1 measurements, given the Reynolds numbers in all but the highest of the pump powers are below the transition regime. However, this is not observed in the data, which shows significant deviations from normal Gaussian distributions and a high degree of fluctuation across pump powers and pipe depth. Two potential explanations present themselves. The change in pipe diameter, along with the associated deceleration and pressure variations destabilizes the flow, causing substantial fluctuations in the velocity measurements. This is seen in the standard deviation results at the low pump powers close to the quiescent layer, where standard deviation is much larger than further into the pipe. In the dilute regime of the antibodies, the scattering signal was much weaker than later results, shown in higher residual difference from the Hagen–Poiseuille model. The large deviation seen in the statistics could additionally be a result of the much weaker signal.

### Flow of High Concentration mAb-1 Solutions

The same mAb-1 sample was concentrated using tangential flow filtration by opening the bypass in Figure 2. A MiniKros Sampler hollow fiber column filter was used with a modified poly­(ether sulfone) media of a 50 kDa rating, with a total surface area of 2600 cm^3^. A pressure differential of ∼1.1 bar was maintained over the column to cause a portion of the flow to pass through the membrane and into an external reservoir while the concentrate recirculated. The final volume of the concentrate was ∼2 L, with the concentration of the concentrate measured as 84.7 ± 0.2 mg mL^–1^ using a SoloVPE.

Velocity profiles were taken at select percentages of pump power as previously. The radial distance from the center of the pipe was recalculated using a slightly larger refractive index of *n* = 1.32 from eq 2, where the concentration increase resulted in a change in refractive angle. The velocity profiles and fits across the radius of the pipe are shown in Figure 5. At lower pump powers the velocity profiles were better described by a power-law fluid model for non-Newtonian flow behavior of complex fluids.^25^ Unlike Newtonian fluids, power-law fluids exhibit a shear-dependent viscosity and the velocity profile of a power-law fluid is given^25^

5 $$v\left(r\right)=\frac{n}{n+1}{\left(\frac{\partial P}{\partial z}\frac{1}{2K}\right)}^{1/n}\left(R^{n+1/n}-r^{n+1/n}\right)$$

where *K* is a flow consistency index, and *n* a dimensionless power exponent that indicates the flow behavior, with *n* < 1 shear thinning and *n* > 1 shear thickening. As the maximum depth of the apparatus does not reach a plateau the data does not outright confirm power-law behavior; reasonable fits can still be made using eq 3 although in all 3 cases of the lower pump powers the better fit was eq 5.

**Figure 5:**
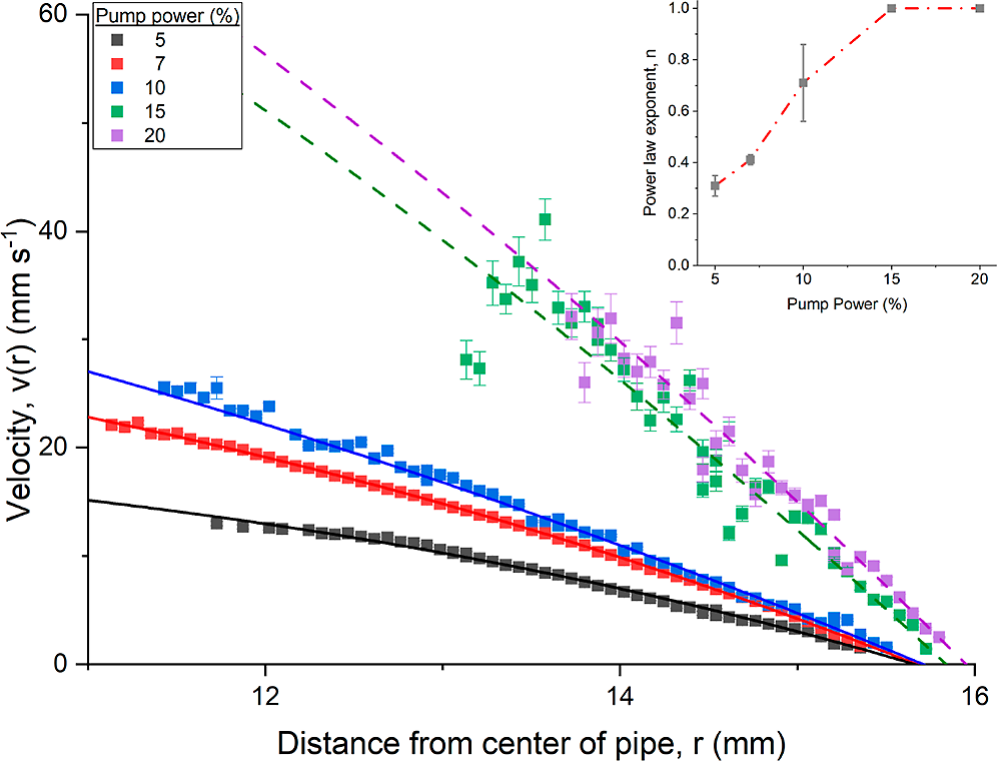
Velocity profile *v*(*r*) as a function of radial depth into the pipe section, *r* of a 85 mg mL^–1^ mAb-1 solution at different pump speeds as a percentage of total power. Dashed lines correspond to a fit of eq 3, with solid lines fits of eq 5. The inset details the power law exponent *n* for different percentages of pump power.

Statistics of the probability density functions of $\frac{v-\mathrm{v̅}}{\mathrm{v̅}}$ at different pump powers and radial distances from the wall of the pipe are shown in Figure 6. The standard deviation shows precise values across different depths at 7 and 10% pump power, or 85 and 120 L h^–1^. A more pronounced U shape with a plateau is seen that follows the same trend as the dilute standard deviation, although better defined. The standard deviations of the velocities in higher pump power measurements are large, which could be a result of transitional flow regime and nonlinear interactions. The large standard deviation for 5% pump power could either be a result of the signal being more susceptible to pump flow at low velocities or, a nonlinear response in the fluid to small shear changes.

**Figure 6:**
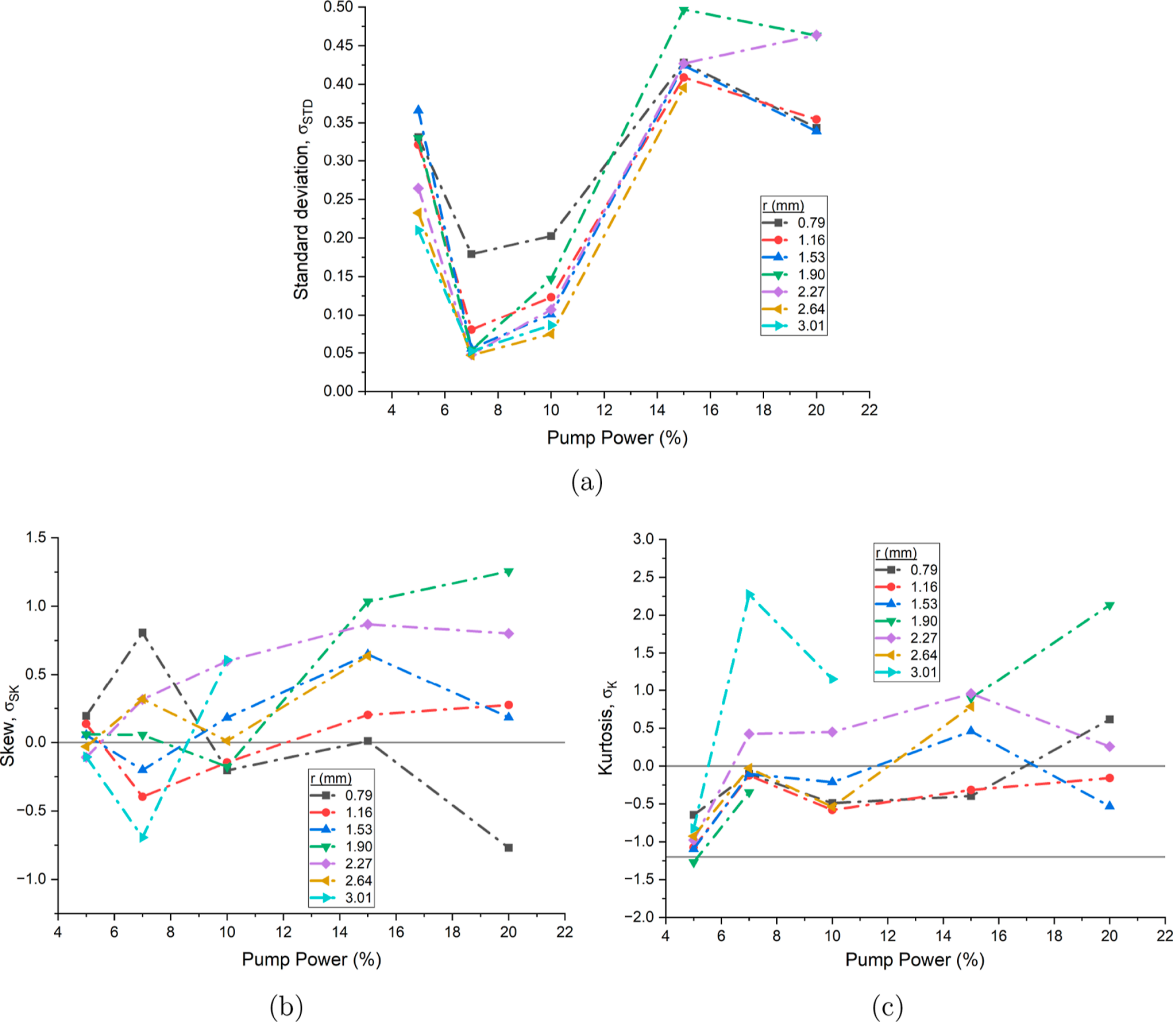
Standard deviation (a), skew (b) and kurtosis (c) of probability density functions of $\frac{v-\mathrm{v̅}}{\mathrm{v̅}}$ as a function of pump power at different positions in the pipe for a 85 mg mL^–1^ mAb-1 solution. Lines at σ_SK_ = 0 for zero skew, σ_K_ = 0 for a normal distribution and σ_K_ = −1.2 for a uniform distribution have been added to aid the viewer.

Both skew and kurtosis show a tendency to increase with pump power, indicating a transition from Gaussian and more uniform velocity distributions at low pump powers toward distributions with more pronounced positive skew and higher kurtosis at higher powers. At 5% pump power, the measurements remain relatively consistent across all radial depths, exhibiting near-zero skew and distributions close to Gaussian. This behavior is expected, as increasing pump power can introduce localized regions of turbulent or unstable flow, where deviations from Gaussian statistics and increased variability in velocity are more likely to occur.^21,26^

For the concentrated mAb-1 sample, it can be concluded that at low shear rates the fluid exhibits a highly structured microenvironment that imparts mild shear-thinning behavior, resulting in a power-law fluid. As shear rate increases, the internal structure gradually breaks down or reorganizes, diminishing the non-Newtonian effects and leading to an increase in *n*. This behavior reflects a transition from a non-Newtonian to a quasi-Newtonian flow regime as shear-induced structural relaxation dominates.

The lower pump powers exhibit noticeably lower residuals from the fits compared to the dilute concentration. A comparison of the different concentrations at 7% pump power is shown in Figure 7a, with similar residual differences observed at 5 and 10%. This suggests either a more stable flow or improved signal quality, resulting in more accurate Gaussian fits of the power spectral density. This would be consistent with a more concentrated sample, where the increased density of scatterers increases the back scattering signal.

**Figure 7:**
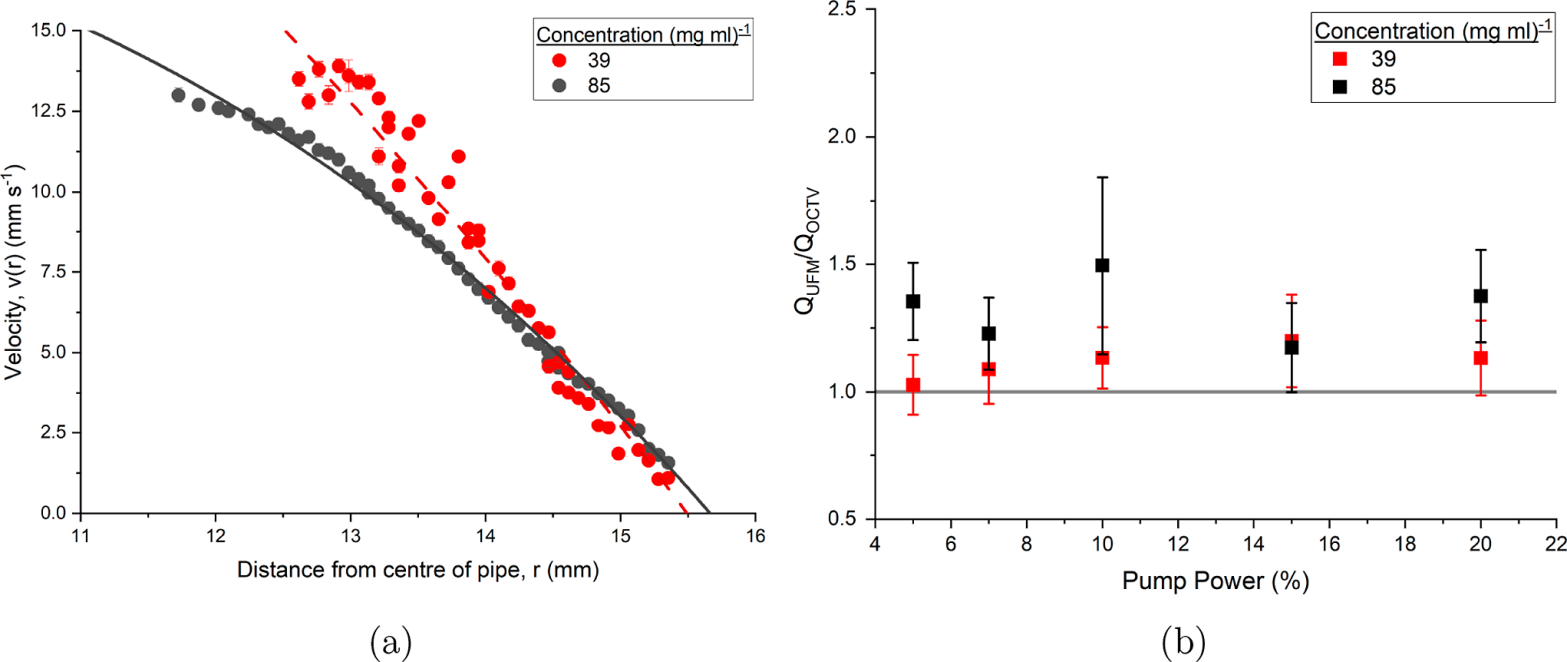
(a) Velocity profile as a function of distance from the center of the pipe at 7% pump power of the 39 mg mL^–1^ and 85 mg mL^–1^ mAb-1 solution. While the pump power is the same, the two fluids exhibited different flow rates due to the different viscosities. (b) Volumetric flow rate comparisons between those taken from the ultrasonic flow meters divided by those derived from the OCTV data. Errors are propagated from both sources, and the line at *Q*_UFM_/*Q*_OCTV_ = 1 indicates full agreement between the sensors.

Figure 7b shows the volumetric flow rate measurements of 39 mg mL^–1^ and 85 mg mL^–1^ mAb-1 solutions as a ratio of the ultrasonic flow meter (UFM) sensors compared to derivations from flow profile fits using OCTV. The flow rates (*Q*) are given from integrating the fits, and then rewritten in terms of *v*_max_. For a Newtonian fluid

6 $$Q=\frac{1}{2}\pi R^{2}v_{\max}$$

and for a power-law fluid

$$Q=\frac{\pi R^{2}v_{\max}\left(n+1\right)}{\left(3n+1\right)}.$$ 7

The errors were propagated from both the quality of the fits and the error in the ultrasonic flow meters. The error from the fits varied in size, with both mAb concentrations showing larger errors at 15 and 20% from larger residuals, larger errors on individual points, and a shallower depth of penetration. The 10% pump power for 85 mg mL^–1^ had a significantly larger error for *n* which is reflected in the error of *Q*_OCTV_. The error in the ultrasonic flow meters was expected to be 10%.

The flow rates derived from OCTV measurements (*Q*_OCTV_) show a consistent underestimation when compared to those obtained via the UFM sensor (*Q*_UFM_), with *Q*_UFM_ typically 1–1.5 times larger. One contributing factor is the presence of 1/*f* noise in the power spectral density, which complicates the accurate detection of the Doppler frequency. To mitigate this, a depth-dependent cutoff frequency is applied to exclude low-frequency noise, but this becomes increasingly more challenging at greater depths where the Doppler signal amplitude weakens. In conditions of weak scattering in dilute mAb solutions, this can suppress the true signal, leading to systematic underestimation of velocity and thus flow rate.

This disparity becomes more pronounced with increasing pump speed at a concentration of 39 mg mL^–1^, aligning with observed reductions in penetration depth and poorer signal fits. Considering the large errors due to the UFM sensors, however, the dilute concentration results are largely within the bounds. At low pump powers with the higher concentration sample, where the solution exhibits power-law behavior, the discrepancy from UFM measurements is greatest. UFM sensors are known to exhibit deviations up to 15% in non-Newtonian fluids, particularly in transitional flow regimes^27^ and systematic errors are introduced due to the varying acoustic attenuation by specific samples. Since the sensors are located within the narrower pipe sections, it is plausible that localized turbulence or transitional flow is introducing error. At 5 and 7% pump power these discrepancies are most prominent and not fully accounted for in the reported uncertainties.

### Flow of mAb-2 Gels

Gelation could be encountered in downstream processing and purification as shown in previous work,^12^ where mAb-2 experienced an acetic acid induced continuous phase transition to a gel. Gelation through this route is fairly rare as many mAbs,^28^ including mAb-1, demonstrate resistance to low pH conditions. However, similar challenges are encountered in other flow-paths involving gels, such as paraffin deposition in crude oil pipelines^29,30^ and clot formation in blood flow.^31^

A 1.0 L solution of mAb-2 at an initial concentration of 52.1 mg mL^–1^ in storage buffer was diluted to a volume of 1.8 L. A final concentration of 28.5 mg mL^–1^ was measured via off-line SoloVPE analysis. The solution was then pumped through the system at varying speeds, expressed as percentages of total pump power. Due to the low protein concentration, the OCTV signal was weak, especially at pump powers above 7%, where penetration depth and signal quality deteriorated significantly. At 7% pump power, shown in Figure 8 (blue data points), the measured penetration depth reached less than 2 mm into the sample. As observed previously with mAb-1 measurements, a zero-velocity boundary layer was present, extending up to approximately 0.4 mm from the pipe wall.^32^

**Figure 8:**
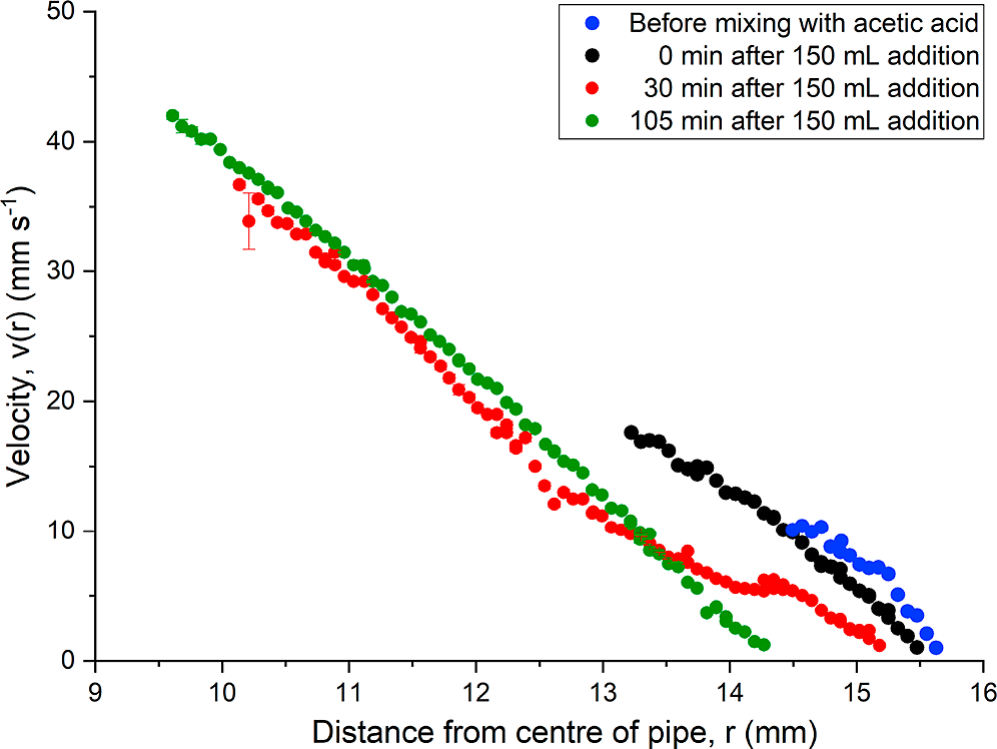
Velocity profile as a function of distance from the center of the pipe of a 29 mg mL^–1^ mAb-2 solution at 7% pump power. The key indicates the length of time since adding 150 mL of acetic acid to the solution - which came after an initial addition of 100 mL acetic acid 16 h before, where limited change in velocity data was observed.

A 75% glacial acetic acid (13 M) solution was slowly mixed with the mAb-2 sample while it was pumped continuously around the flow path. To ensure adequate mixing, 100 mL of acetic acid was gently pipetted into the suspension over a 30 min period to prevent localized concentration gradients. This resulted in a final molarity of approximately 0.68 M and slightly diluted the mAb suspension further. Throughout the addition and mixing stage, the pump power was maintained at 7% to ensure continuous circulation over the large sample. No significant change in the velocity profile or solution was observed after the initial mixing of 100 mL. The sample was left circulating at 7% pump power for 16 h after which an additional 150 mL of 75% glacial acetic acid was pipetted over a 40 min window, for a final volume of 250 mL and a concentration of 1.44 M. Velocity profiles were taken directly after the final addition and shown in Figure 8, where the time represents the curing time after the final addition of acetic acid. Acquiring complete velocity profiles can take up to 15 min depending on the maximum penetration depth; in fluids undergoing a transitional processes, this may fail to capture the full state of the system at a given time point. Additional velocity measurements after *t* = 135 min showed no further changes to the flow profile.

Gel deposition behavior was observed in the evolving flow profiles, where a zero-velocity boundary layer extended progressively to a maximum of 1.7 mm by the 105 min mark. Comparable behavior has been observed in crude oil pipelines, where paraffin deposition produces a similar build-up requiring mechanical removal.^30^ Fluids traveling over a cavity exhibit a similar velocity profile to the data at 30 min,^33,34^ where a secondary velocity peak emerges due to the frictional forces from a stagnant region. In the flow-path, the diffuser and consequent increase in pipe radius could be enforcing this cavity. The evolving viscoelastic nature of the gelling mAb-2 solution likely amplifies this effect, as the increased viscosity near the walls and onset of yield stress behavior may inhibit smooth expansion and promote flow separation and gel formation.

Both the maximum depth of measurement and the signal quality increased as the gel progressed, where the radius of scatterers increased in size over the course of the experiment. The flow rate decreased from *Q* = 103 L h^–1^ before the addition of acetic acid to *Q* = 76.3 L h^–1^ after the full transition, where the former was calculated through the Hagen–Poiseuille fit and the latter treated as a power-law fluid, demonstrating an increase in viscosity over the length of the pipe. Exact flow rate comparisons with the ultrasonic flow meters were not possible for this set of experiments, as a small amount of bubbles entered the flow due to turbulence in the sample analysis chamber, and could not be adequately removed due to the elastic nature of the fluid. This likely had a minor effect on the results, where bubbles act as source of turbulence and multiphase flow.^35^

Additional measurements were performed to demonstrate time-dependent shear behavior in the flow, shown in Figure 9a. Following on from the final measurement at *t* = 135 min, the pump was halted for 30 min. The flow was returned to 7% and a velocity profile taken after the hold. The pump was then set to 40% power for 30 min and returned to 7% where another flow profile was taken, before holding for a further 30 min and a final velocity profile at 7%. A memory response in the sample was observed. When fitting a power-law fluid model, the flow rate decreases from the high to no-shear results revealing a time dependent viscosity and a thixotropic/antithixotropic effect, where no-shear reduces the flow and high-shear returns the flow to its previous condition. The gel boundary layer also changes in depth from the pipe wall.

**Figure 9:**
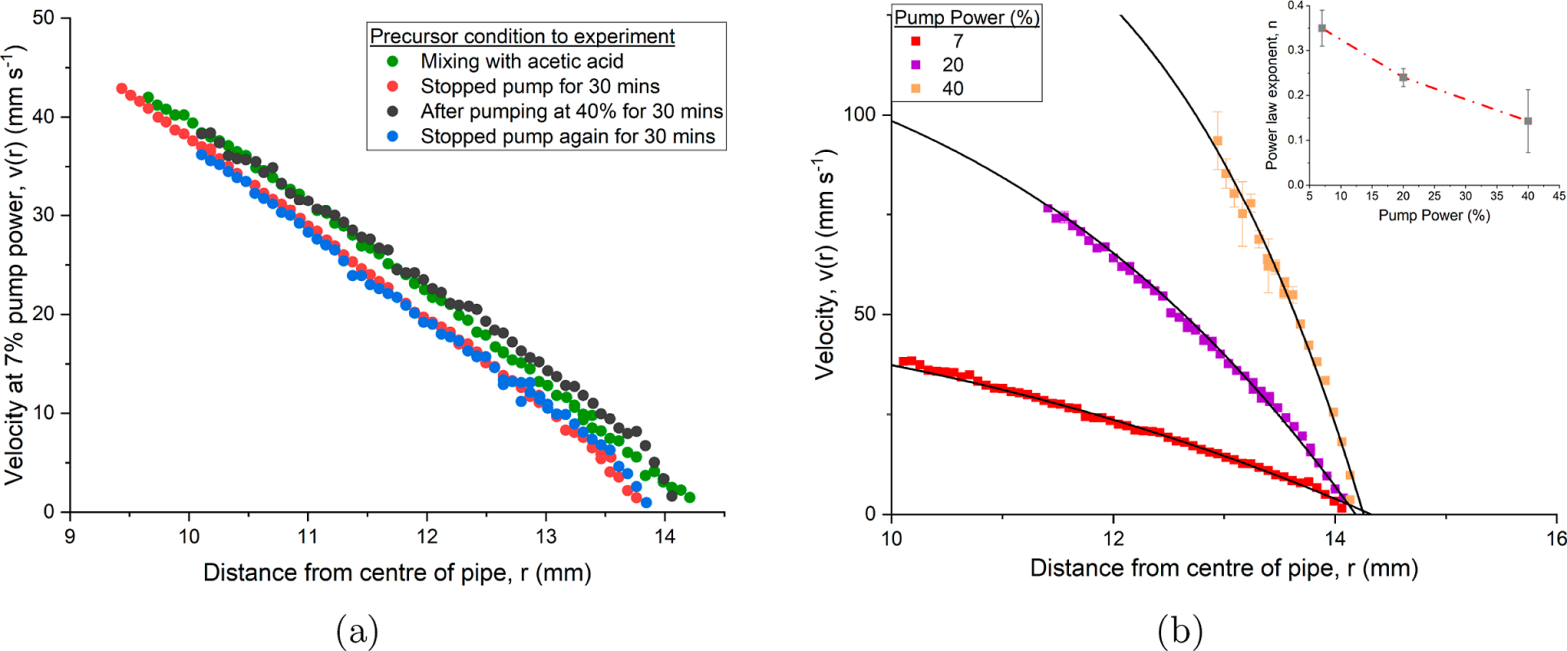
(a) Velocity profile as a function of distance from the center of the pipe at 7% pump power, with different conditions leading to the velocity measurements. The green data points correspond to the green data set in Figure 8, and the conditions were followed in order of the key. (b) Velocity profile as a function of distance from the center of the pipe for different pump powers of the mAb-2 solution postgel. Solid lines are fits of eq 5, where the inset shows the power law exponents of these fits.

In Figure 9b, full velocity profiles of the mAb-2 acetic acid solution are plotted as a function of distance from the center of the pipe at 7, 20 and 40% pump power. Fits were made using the power-law fluid model from eq 5 and the exponents for each pump speed are seen in the inset. The results show a decrease in the power-law exponent and size of the velocity plateau at higher pump powers, or higher shear rates. The change in power-law exponents is indicative of the progressive alignment and disentanglement of the protein chains under shear, leading to a reduction in apparent viscosity. As the shear rate increases, the fluid transitions from a highly structured state to a more flow-aligned configuration, effectively lowering the resistance to deformation and thus decreasing the value of *n*. Similar behavior is seen in other biopolymers such as cellulose.^36^ There is limited change in the gel deposition layer at higher shears, only diminishing by ∼0.2 mm between 7 and 40%, similar to the results shown in Figure 9a, demonstrating a resistance to shear when compared to the zero-velocity boundary layers in both the dilute and concentrated mAb-1 samples.

Statistics of the flow are shown in Figure 10. Here the standard deviation, skew and kurtosis of the probability density functions of $\frac{v-\mathrm{v̅}}{\mathrm{v̅}}$ are compared at different depths and pump powers. Standard deviation is initially high before decreasing with increasing pump power. At lower powers the perturbation of the pump is more noticeable and corresponds to a larger variation in velocity measurements. The results show correlation with depth into the pipe, where standard deviation generally decreases, more noticeable at the lower power of the pump. The skew is negative at the closest point to the gel boundary layer at *r* = 1.90 mm, before reaching the most positive at the second distance of *r* = 2.27 mm, then decreasing to neutral levels with pipe depth. The skew can be seen here as an effect of the velocity gradient;^26,37^ the negative skew and large standard deviation corresponds with a drag of the distribution toward the sedimentary gel layer at low depths, the positive skew as the velocity increases at the fastest rate with pipe depth, and neutral where the rate of change in velocity over the pipe slows.

**Figure 10:**
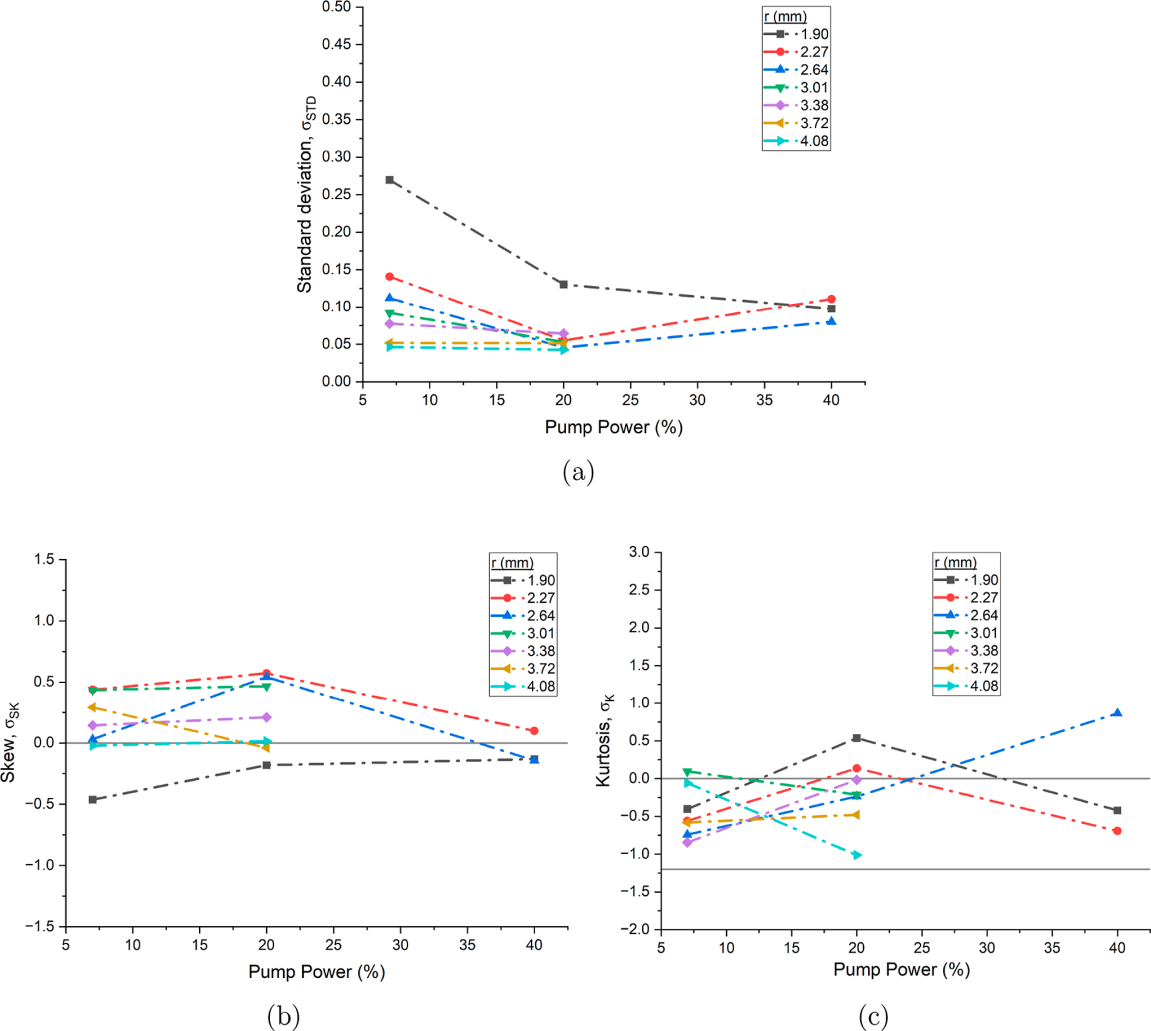
(a) Standard deviation, (b) skew and (c) kurtosis of probability density functions of $\frac{v-\mathrm{v̅}}{\mathrm{v̅}}$ as a function of pump power at different positions in the pipe for mAb-2. Lines at σ_SK_ = 0 for zero skew, σ_K_ = 0 for a normal distribution and σ_K_ = −1.2 for a uniform distribution have been added to aid the viewer. The *y*-axis is rescaled to match Figures 4 and 6 for ease of comparison.

The velocity measurement fluctuations are minimal, as demonstrated by the low residuals from the power-law fluid fits shown in Figure 9b. Consequently, the statistical analysis of the velocity profiles primarily reflects the intrinsic flow dynamics rather than noise or artifacts arising from the measurement apparatus. These results are consistent with laminar flow conditions throughout, exhibiting near-Gaussian velocity distributions and low standard deviations across all pump powers.^21^ This behavior further suggests a significantly higher effective viscosity compared to mAb-1, which is consistent with the formation of a protein hydrogel.

## Conclusion

A bespoke optical coherence tomography velocimeter was demonstrated for the first time with downstream processing of biologics; specifically pipe flow of high concentration monoclonal antibodies and BSA (Supporting Information). For mAb-1, both Newtonian and power-law flow behavior were observed under dilute and concentrated conditions, respectively. These velocity profiles were compared with flow rates obtained from clamped ultrasonic flow meters and reasonable agreement was observed in the dilute regime. At higher mAb concentrations, improved fits were obtained using a power-law model, where the flow index *n* increased with pump power, approaching Newtonian behavior at high pump rates. This suggests the breakdown of an internal microstructure within the fluid under high shear. Discrepancies between UFM-derived and OCTV-derived flow rates were more pronounced in the concentrated samples, consistent with known limitations of UFMs with non-Newtonian samples.^27^

Statistical analysis of transient velocity measurements did not reveal clear evidence of turbulence in dilute solutions. Instead, high noise levels were measured in the velocity fluctuations, attributed to weak scattering of the low turbidity samples. In contrast, concentrated solutions with higher scattering intensity enabled more stable velocity profile extraction and improved fit quality. The literature remains limited on the use of OCTV for dilute, transparent colloidal suspensions; results here demonstrate deeper penetration into the flow field, albeit at the expense of resolution. These trends scale with concentration, consistent with an increased number density and hydrodynamic radius of scatterers.

Novel flow behavior was observed using OCTV in a fluid undergoing a sol–gel phase transition, where mAb-2 aggregated under low pH conditions induced by mixing with acetic acid. Real-time measurements revealed gel deposition near the pipe boundary, characterized by the emergence and eventual disappearance of a secondary velocity peak. This phenomenon shows parallels with paraffin deposition in crude oil transport^29,30^ and fibrillar aggregation during blood clotting.^31^ The flow exhibited time-dependent viscosity and shear history dependence, as evidenced by changes in velocity profiles at fixed pump powers. The fluid conformed to a power-law model with a decreasing flow index *n* as pump power increased, indicating progressive structural alignment under shear. Statistical analysis confirmed laminar flow conditions with predominantly Gaussian velocity distributions, while deviations in skewness highlighted sharp velocity gradients associated with the evolving boundary layer.

OCTV was successfully demonstrated for real-time, in-line velocity analysis of dilute and concentrated mAb solutions, as well as a mAb sample undergoing gelation. This enabled direct insights into flow behavior across a range of regimes. Integration of pressure sensors, would further enable in-line viscosity estimation. Incorporating an electro-optic modulator into the interferometer setup could suppress the characteristic 1/*f* noise,^38^ thereby enhancing sensitivity to low velocities and improving the accuracy of flow rate measurements. Such improvements would reduce errors arising from misfitting of Doppler peaks, which may explain discrepancies between OCTV-derived and ultrasonic flow sensor data. The Doppler phase shift is also influenced by the diffusion coefficient, with smaller particles yielding broader spectral peaks and higher velocity uncertainty. This property could be exploited for in-line particle sizing.^39^ Further extensions of the technique, such as scanning DLS-OCT, offer additional opportunities for size characterization.^40^

## Supplementary Material



## Notes

Velocity profile of a bovine serum albumin (ZIP)
