# Supplementary figures and images for: Optical Coherence Tomography Velocimetry for In-Line Processing of Biologics: Concentrated and Gelling Monoclonal Antibody Solutions
Source: ACS Eng Au. 2026 Feb 24;6(2):296–306. doi: 10.1021/acsengineeringau.5c00083 (PMC13088178; doi:10.1021/acsengineeringau.5c00083)

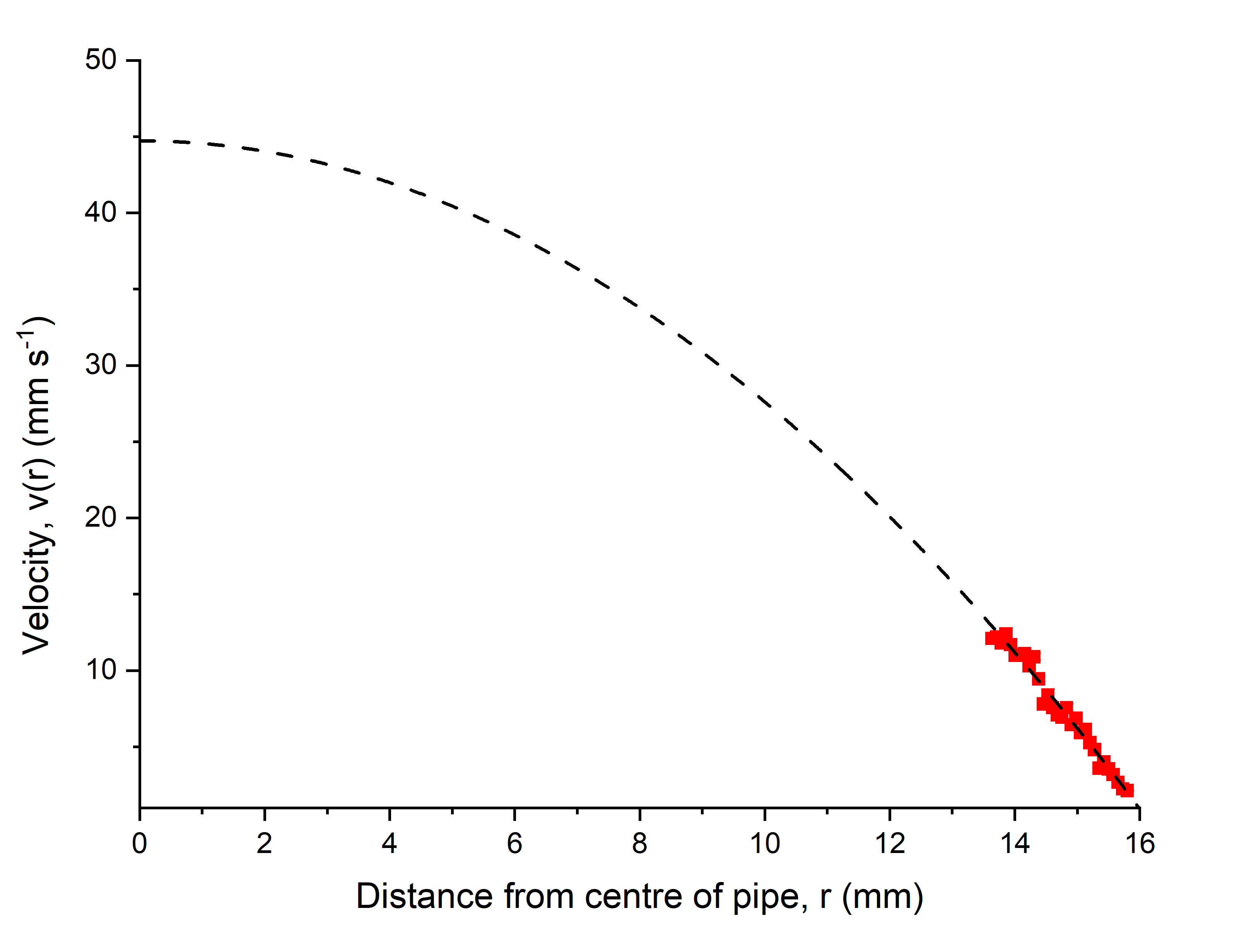

Supplement: Supplementary file 1 [file eg5c00083_si_001.zip › BSA.png]
